# Optimizing Lung Cancer Diagnostics: Insights from a Fast-Track Program in a Complex Healthcare Setting †

**DOI:** 10.3390/life15030362

**Published:** 2025-02-25

**Authors:** Paolo Scanagatta, Alessandro Bertolini, Giuseppe Naldi, Francesca Antoniazzi, Francesco Inzirillo, Casimiro Eugenio Giorgetta, Eugenio Ravalli, Gianluca Ancona, Sara Cagnetti, Claudio Barbonetti, Fabiano Stangoni

**Affiliations:** 1 Division of Thoracic Surgery, Morelli Hospital, ASST Valtellina e Alto Lario, 23035 Sondalo (SO), Italy; giuseppe.naldi@asst-val.it (G.N.); francesco.inzirillo@asst-val.it (F.I.); casimiro.giorgetta@asst-val.it (C.E.G.); eugenio.ravalli@asst-val.it (E.R.); gianluca.ancona@asst-val.it (G.A.); sara.cagnetti@asst-val.it (S.C.); 2 Department of Oncology, ASST Valtellina e Alto Lario, 23100 Sondrio (SO), Italy; alessandro.bertolini@asst-val.it (A.B.); francesca.antoniazzi@asst-val.it (F.A.); fabiano.stangoni@asst-val.it (F.S.); 3 Division of Nuclear Medicine and Radiotherapy, Department of Clinical Services, ASST Valtellina e Alto Lario, 23100 Sondrio (SO), Italy; claudio.barbonetti@asst-val.it; 4 Specialization School of Oncology, Statale University of Milan, 20122 Milano (MI), Italy

**Keywords:** fast track, lung cancer, diagnostic pathways, multidisciplinary team, case manager, early diagnosis, oncology workflow, healthcare efficiency, lung cancer screening, health system optimization

## Abstract

Lung cancer remains a leading cause of cancer-related mortality, with diagnostic delays significantly impacting patient outcomes. Despite advancements in diagnostic strategies, inefficiencies persist, particularly in geographically complex regions with limited healthcare resources. The Fast-Track Program was developed to address these challenges in lung cancer diagnostics within the geographically complex and resource-limited Valtellina region. This prospective observational study compared patients managed under the Fast-Track pathway (May–August 2024) with those following standard diagnostic procedures (January–April 2024). The program integrated structured, pre-scheduled diagnostic slots, a rotating Case Manager role, and weekly multidisciplinary team (MDT) discussions to enhance coordination and reduce diagnostic timelines. Results showed a significant reduction in the mean time to definitive diagnosis from 42.9 days (95% CI: 35.6–50.3) in the control group to 25.0 days (95% CI: 20.8–29.3) in the Fast-Track cohort (*p* < 0.001). Patient adherence to diagnostic pathways improved from 71% to 92% (*p* < 0.05), while satisfaction scores increased from 64% to 89%, with patients rating their experience as “very good” or “excellent” (*p* < 0.05). Although the predefined clinical significance criteria were not fully met, the program demonstrated a favorable trend toward improved efficiency and patient-centered care. These findings support the feasibility and scalability of structured diagnostic workflows in streamlining lung cancer diagnostics, with potential implications for broader oncological and chronic disease management in resource-constrained healthcare settings.

## 1. Introduction

Lung cancer remains the leading cause of cancer-related mortality worldwide, accounting for 35,700 deaths in Italy in 2022, with an estimated 44,000 new cases in 2023 [1]. Despite advances in diagnostic technologies and treatment strategies, lung cancer outcomes remain suboptimal, largely due to persistent delays in diagnosis, which hinder early intervention and compromise survival rates. Recent studies have emphasized that prolonged diagnostic pathways not only reduce therapeutic effectiveness but also lead to increased healthcare costs and patient distress [2,3].

**Figure 1:**
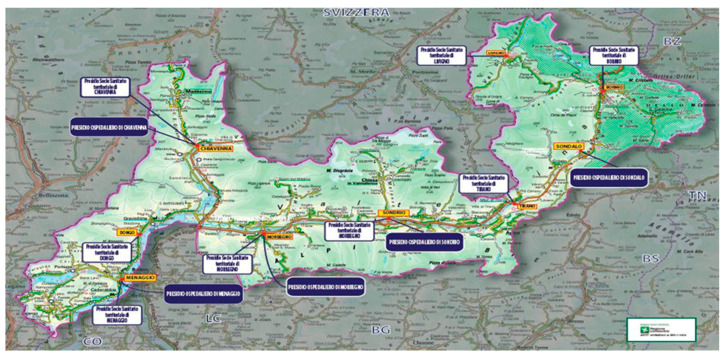
Geographical and demographic context of the Valtellina region. The Valtellina region is entirely mountainous, extending from the Lepontine Alps to the Eastern Rhaetian Alps, including the northern slopes of the Orobie Prealps. The region covers approximately 3795 km^2^, with a total length of 373 km from its westernmost to easternmost points. The resident population consists of nearly 197,000 inhabitants, spread across small villages and towns, ranging from less than 100 residents in some municipalities to approximately 23,000 in the main city, Sondrio. The region experiences notable seasonal population increases due to continuous tourist flows, particularly during winter and summer. Due to the region’s geographic dispersion and challenging road network, strategic healthcare planning is essential to ensure equitable access to medical services. To address these challenges, the Azienda Socio-Sanitaria Territoriale (ASST) of Valtellina and Alto Lario implemented the Fast-Track Program, an innovative diagnostic model aimed at reducing diagnostic delays, optimizing resource allocation, and improving patient outcomes. The program integrates predefined diagnostic pathways and introduces a Case Manager to oversee and coordinate the diagnostic process, ensuring effective communication among general practitioners (GPs), hospital specialists, and territorial healthcare providers. Beyond its local implementation, this structured model holds potential for broader applications in other regions facing similar logistical and healthcare access challenges. By integrating a standardized workflow with multidisciplinary coordination, the Fast-Track Program serves as a scalable framework that could enhance cancer diagnostics in decentralized healthcare systems.

The Valtellina region, a fully mountainous area spanning 3795 km^2^ with a total length of 373 km, faces geographical and logistical barriers that complicate access to healthcare. Its dispersed population of nearly 197,000 inhabitants is spread across small municipalities, with notable seasonal variations due to tourism influxes (Figure 1). Beyond geographical challenges, the region experiences a chronic shortage of specialized medical personnel. The proximity to Switzerland, where salaries are 2–3 times higher, has led to significant workforce migration, making it difficult to recruit and retain key specialists, including pulmonologists, thoracic surgeons, interventional radiologists, and pathologists. This also affects primary care, as the number of general practitioners (GPs) is progressively declining, leading to longer waiting times for initial diagnostic referrals. These challenges necessitate tailored healthcare strategies to ensure equitable access, particularly for lung cancer diagnostics. In fact, these inefficiencies create a high risk of diagnostic dispersion, redundant consultations, and prolonged patient uncertainty, all of which contribute to delays in both diagnosis and treatment [4,5]. Fast-Track models have emerged as effective strategies to mitigate these delays, with structured care pathways showing success in various healthcare systems, including those in the UK, Denmark, and Spain. These models have demonstrated a reduction in time to diagnosis and treatment initiation, underscoring the need for regionally adapted approaches to optimize lung cancer management [6,7].

The question of cost-effectiveness is central to the evaluation of Fast-Track models. While previous studies have highlighted the financial benefits of reducing unnecessary hospital visits and redundant tests, a comprehensive cost-analysis of the Fast-Track Program is still needed. Preliminary data suggest that reducing diagnostic delays could lead to economic savings by minimizing disease progression-related expenses and streamlining resource utilization, but further studies are warranted to quantify these effects [8,9].

A key feature of the program is its multidisciplinary approach, which involves weekly case discussions among thoracic surgeons, oncologists, radiologists, pathologists, and other specialists to ensure that each patient follows an optimized diagnostic and therapeutic pathway. The program also incorporates reserved diagnostic slots for lung cancer patients, minimizing bottlenecks in access to imaging, endoscopic procedures, and biopsies, while mitigating delays caused by geographical and logistical constraints [10,11,12,13].

This study presents the design, implementation, and impact of the Fast-Track Program, evaluating its effectiveness in streamlining lung cancer diagnostics and assessing its potential applicability in other complex healthcare settings. The findings underscore the importance of a structured, multidisciplinary, and patient-centered approach in optimizing lung cancer diagnosis and improving overall healthcare efficiency.

## 2. Materials and Methods

The Fast-Track Program was developed to address critical challenges in the diagnostic management of lung cancer patients in the Valtellina region. Its design integrated clinical and organizational innovations aimed at reducing diagnostic delays and ensuring standardized patient care pathways.

This is a prospective sequential observational study comparing two cohorts: patients managed under the Fast-Track Program and those under standard diagnostic pathways. The selection of the two cohorts was based on a temporal criterion rather than an active selection process. Patients were assigned to the standard diagnostic pathway (January–April 2024) before the implementation of the Fast-Track Program, while those evaluated after the program’s introduction (May–August 2024) were automatically managed under the new model. This sequential observational approach allowed for a natural comparison of diagnostic timelines and adherence to pathways without introducing selection bias. The study was approved by the ASST Valtellina e Alto Lario administration. Since it involves organizational aspects of standard clinical practice, without modifications to diagnostic tests or therapeutic approaches, prior approval from the Ethics Committee or Institutional Review Board was not deemed necessary. All patients provided informed consent for the anonymous use of their data. The study was conducted in accordance with the principles of the Declaration of Helsinki.

1. Program Design

**Figure 2:**
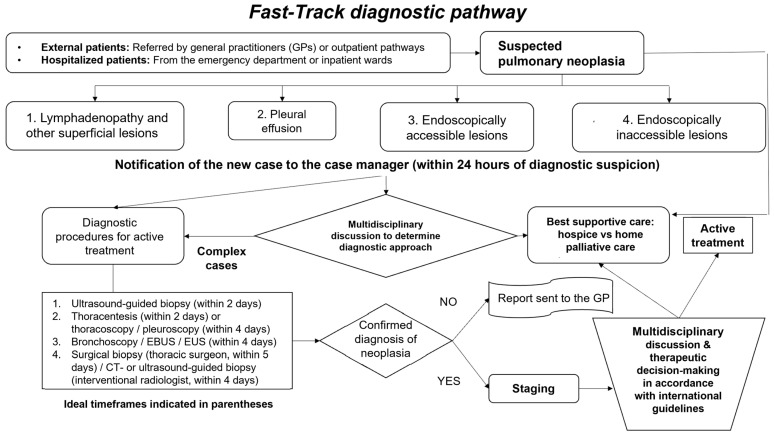
Fast-Track diagnostic pathway. This flowchart outlines the structured Fast-Track diagnostic pathway, beginning with patient referral and coordination by the Case Manager. It categorizes diagnostic scenarios, integrates multidisciplinary team (MDT) collaboration, and ensures timely diagnosis while optimizing the decision-making process for further management. This pathway aligns with the recommendations followed in the Lombardy region (to which Valtellina belongs) as outlined in the PDTA (Percorso Diagnostico Terapeutico Assistenziale) for lung cancer by our institution.

The program’s core feature was the reservation of dedicated diagnostic slots for suspected lung cancer patients. These slots included imaging studies such as contrast-enhanced computed tomography (CT) and fluorodeoxyglucose-positron emission tomography (FDG-PET), as well as invasive diagnostic procedures like bronchoscopy and biopsies [2,10]. This approach ensured that patients could undergo essential tests within a predefined timeframe, notably reducing waiting periods. Although precise historical data on pre-existing diagnostic timelines in our region are not available, the Fast-Track Program was implemented following evidence from similar initiatives in other settings. The study design allows for a prospective sequential observational analysis, comparing a cohort of patients managed under standard diagnostic pathways before the program’s introduction with those evaluated after its implementation. Figure 2 provides a visual representation of the structured Fast-Track diagnostic pathway, outlining key stages from patient referral to multidisciplinary team collaboration.

2. Case Manager Role and Patient Workflow

Unlike traditional Fast-Track programs with a single coordinator, this program adopted a rotational Case Manager system within the MDT to ensure continuous patient oversight, preventing workflow disruptions due to individual availability constraints. Given that each specialist is also engaged in other clinical activities, a rotational model minimizes the risk of workflow disruptions due to absences or scheduling conflicts, thereby preserving the efficiency gains of the Fast-Track approach. Moreover, this approach ensured that each patient had a dedicated specialist managing their case at every stage of the diagnostic process. The workflow was structured on a four-day cycle, with daily rotation of responsibilities among MDT specialists.

Each day, an MDT specialist assumed responsibility for newly referred patients, integrating them into the Fast-Track pathway and scheduling initial diagnostic tests. Coordination rotated daily, ensuring continuous oversight of ongoing cases and seamless integration of new referrals. This system ensured that all patients progressed efficiently through the diagnostic process, minimizing delays and ensuring adherence to evidence-based pathways; when weekends or public holidays interrupt the cycle, the designated Coordinator must ensure that newly referred cases are processed by the end of the second subsequent working day.

This structured workflow ensures continuity of care, rapid case assessment, and optimal resource utilization. The rotational MDT system minimizes diagnostic delays and ensures that each patient adheres to an evidence-based diagnostic pathway.

3. Multidisciplinary Approach

A multidisciplinary approach was a fundamental component of the Fast-Track Program, ensuring coordinated and timely decision-making for patients with suspected lung cancer [6]. Weekly multidisciplinary team (MDT) meetings were implemented to assess cases, define optimal diagnostic strategies, and facilitate seamless transitions between diagnostic and therapeutic phases. These meetings included specialists from Thoracic Surgery, Pulmonology, Medical Oncology, Radiology, Pathology, and Radiotherapy, with the possibility of involving Interventional Radiology, Nuclear Medicine, and other specialists (e.g., Neurosurgery and General Surgery) depending on the complexity of individual cases. The Case Manager played a central role in facilitating MDT discussions, providing regular updates on patient progress, coordinating diagnostic pathways, and ensuring adherence to the established workflow. The MDT meetings held formal medical–legal validity, requiring the presence of at least one pulmonologist, thoracic surgeon, oncologist, radiotherapist, and radiologist to validate decision-making and ensure compliance with regulatory standards. The multidisciplinary discussion provided several advantages. It enhanced the appropriateness of the diagnostic–therapeutic pathway, particularly in complex cases requiring multimodal approaches. By integrating specialists’ expertise, the MDT allowed for the precise selection of diagnostic tests and treatment modalities, minimizing unnecessary procedures and ensuring a more tailored approach to each patient. Additionally, the administrative and organizational aspects of patient management were significantly optimized. The meetings facilitated the immediate exchange of medical records, referrals, and test requests, thereby reducing bureaucratic delays and expediting the diagnostic process. To overcome Valtellina’s geographical challenges, MDT meetings were held remotely using telemedicine platforms. This approach eliminated the need for specialists to travel between different hospital sites, reducing logistical barriers and optimizing discussion times [4]. The healthcare system’s digital infrastructure enabled real-time sharing of imaging, pathology reports, and laboratory diagnostics, ensuring seamless coordination among the involved disciplines and reinforcing a patient-centered, efficient diagnostic model.

4. Study Population and Inclusion Criteria

The program targeted patients with suspected lung cancer identified through referrals from general practitioners, emergency departments, or hospital specialists. Exclusion criteria included hospitalized patients already under the care of thoracic surgeons or oncologists, as their diagnostic pathways were managed within the hospital framework [8]. Eligible patients were enrolled through a streamlined referral system, typically initiated via email communication with the Case Manager. The baseline characteristics of the study population, including demographic data, clinical features, and initial diagnostic parameters, are summarized in Table 1.

5. Diagnostic Pathways

Four primary clinical-radiological scenarios were identified to guide diagnostic workflows:

○ Lymphadenopathy and superficial lesions: These include the use of fine-needle aspiration (FNA) or core biopsy under ultrasound or CT guidance [5]. Lymphadenopathy, in this context, refers to enlarged mediastinal or hilar lymph nodes, which may be secondary to inflammatory, infectious, or malignant processes. In suspected lung cancer cases, differentiating between benign and malignant lymphadenopathy is crucial for proper staging and treatment planning. The diagnostic approach follows national (AIOM) and international (ESMO) guidelines, incorporating endobronchial ultrasound (EBUS) for mediastinal assessment and CT-PET correlation to guide biopsy decisions when necessary (AIOM Guidelines [10]—https://www.aiom.it/linee-guida-aiom-2024-neoplasie-del-polmone/ (accessed on 12 February 2025), ESMO Guidelines—https://www.esmo.org/guidelines/guidelines-by-topic/esmo-clinical-practice-guidelines-lung-and-chest-tumours (accessed on 12 February 2025)). TNM staging was performed according to the 8th edition of the AJCC/UICC guidelines. We also clarified that staging investigations included contrast-enhanced CT, FDG-PET, and, when necessary, endobronchial ultrasound-guided fine-needle aspiration (EBUS-FNA) or mediastinoscopy.
○ Pleural effusion: These include the use of diagnostic thoracentesis or pleuroscopy with biopsy when cytology was inconclusive [5,9].
○ Endoscopically accessible lesions: These include the use of bronchoscopy or endobronchial ultrasound (EBUS) for tissue sampling [5,8].
○ Lesions requiring advanced imaging or surgical biopsy: These include the use of CT-guided percutaneous biopsy or video-assisted thoracoscopic surgery (VATS) [10].

The Fast-Track Program implemented standardized diagnostic pathways based on predefined clinical-radiological scenarios (Table 2). Each scenario was tailored to ensure timely and appropriate diagnostic interventions, with workflows designed to minimize procedural delays.

6. Evaluation Metrics

**Figure 3:**
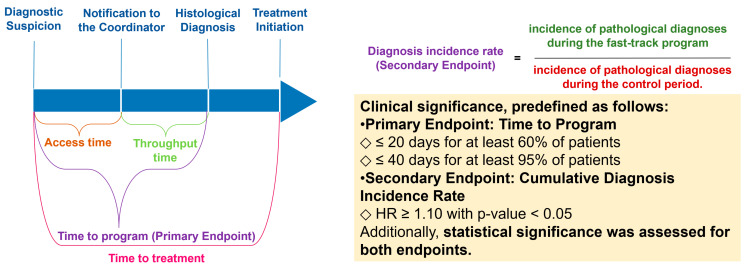
Monitoring the implementation of the Fast-Track Program: efficiency indicators and outcomes. This figure illustrates the key efficiency indicators used to evaluate the Fast-Track Program, including diagnostic timelines and incidence rate comparisons. The primary endpoint is the time to program, defined as the duration from the initial suspicion of lung cancer to histological confirmation or the decision not to proceed with biopsy. The secondary endpoint is the Cumulative Diagnosis Incidence Rate, assessed through hazard ratio (HR) analysis.

A comparative analysis was conducted between two consecutive patient cohorts: those managed under standard care pathways (January–April 2024) and those enrolled in the Fast-Track Program (May–August 2024). The program’s effectiveness was assessed using key performance indicators (KPIs), including time to definitive diagnosis, adherence to diagnostic pathways, and patient satisfaction. The Fast-Track Program was assessed using predefined efficiency indicators and clinical significance thresholds, as summarized in Figure 3 and Table 3. Statistical significance was evaluated using a pre-set threshold of α = 0.05. In addition, clinical significance was predefined based on specific criteria for the primary and secondary endpoints. The primary endpoint, “program time”, was considered clinically significant if ≤20 days was achieved in at least 60% of patients or ≤40 days in at least 95% of cases. The secondary endpoint, “Cumulative Diagnosis Incidence Rate”, was considered clinically significant if HR ≥ 1.10 with *p* < 0.05, indicating a meaningful increase in diagnostic volume. Predefined statistical analyses included chi-square tests for categorical variables; a Fisher’s exact test for categorical data with small, expected frequencies; and a Student’s *t*-test for continuous variables to compare diagnostic timelines between groups.

This structured and patient-centric approach aimed to streamline the diagnostic phase, enhancing both the efficiency of care delivery and patient outcomes.

## 3. Results

A comparison of baseline demographic and clinical characteristics between the Fast-Track Program and control cohorts is presented in Table 1. The implementation of the Fast-Track Program demonstrated notable improvements in diagnostic efficiency and care coordination for patients with suspected lung cancer. The analysis focused on time to definitive diagnosis, adherence to diagnostic pathways, and patient satisfaction.

### 3.1. Reduction in Diagnostic Timelines

Patients in the Fast-Track Program obtained a definitive diagnosis significantly faster than those in standard care pathways. The mean time to diagnosis was reduced from 42.9 days (95% CI: 35.6–50.3) in the control cohort to 25 days (95% CI: 20.8–29.3) in the Fast-Track cohort (*p* = 0.0001). Additionally, the proportion of patients diagnosed within 40 days increased from 49% in the control group to 81% in the Fast-Track cohort, and those diagnosed within 20 days rose from 32% to 53%. While the predefined clinical significance threshold for program time (≤20 days for 60% or ≤40 days for 95%) was not fully met, the observed trend strongly suggests a meaningful improvement in diagnostic efficiency (see Table 3, Table 4, Table 5 and Table 6).

### 3.2. Improved Adherence to Diagnostic Pathways

The introduction of a Case Manager ensured higher adherence to established diagnostic protocols. Among Fast-Track patients, 93% underwent all recommended diagnostic procedures without deviation, compared to 67% in the standard care cohort. This improvement was attributed to the predefined diagnostic slots and the active role of the Case Manager in coordinating patient pathways. The low over-referral rate further validated the program’s efficiency in resource utilization (Table 4 and Table 5).

### 3.3. Impact of Multidisciplinary Team (MDT) Integration

The implementation of the Fast-Track Program emphasized a structured multidisciplinary team (MDT) approach, ensuring real-time collaboration among specialists from Thoracic Surgery, Pulmonology, Medical Oncology, Radiology, Pathology, and Radiotherapy. This strategy streamlined diagnostic workflows and improved adherence to standardized pathways. Figure 3 illustrates the structured monitoring approach used to track program implementation and MDT efficiency.

To enhance diagnostic efficiency and inter-hospital coordination, key performance indicators were monitored, including adherence to diagnostic pathways and timely completion of required workups. These efficiency metrics and adherence rates are summarized in Table 4.

Furthermore, patient satisfaction was evaluated to measure the perceived benefits of the Fast-Track approach compared to standard pathways. A significant improvement in patient-reported satisfaction was observed, as detailed in Table 5.

One of the critical objectives of the program was to increase the incidence rate of new confirmed lung cancer diagnoses by optimizing patient selection and prioritization within the diagnostic process. However, the results indicate that the incidence rate of new lung cancer diagnoses did not show a statistically significant increase in the Fast-Track cohort compared to the control group (HR = 0.70 and *p* = 0.14 in Table 6). While the reduction in diagnostic timelines achieved a statistical significance, it did not fully meet the predefined clinical significance threshold. In medical statistics, it is well established that statistical significance alone does not necessarily translate into meaningful clinical benefit. For this reason, following contemporary trends in clinical research, we defined a priori a primary and a secondary outcome, both of which must be met to confirm a true and incontrovertible advantage for the patient. In our study, although the Fast-Track Program significantly reduced diagnostic delays, further refinements in scheduling and workflow efficiency are necessary to achieve the predefined clinical impact.

These findings are visually summarized in Figure 3, highlighting key efficiency indicators of the Fast-Track Program. While the incidence rate of confirmed lung cancer diagnoses did not show a statistically significant increase, the observed trend suggests an improvement in workflow efficiency and diagnostic prioritization.

### 3.4. Patient Satisfaction and Feedback

Patient surveys confirmed a significant improvement in satisfaction levels with the Fast-Track Program. Specifically, 90% of patients rated their experience as ’very good’ (48%) or ’excellent’ (42%), compared to 57% in the standard pathway cohort, where only 32% and 25% rated their experience as ’very good’ or ’excellent’, respectively. The main benefits cited were reduced waiting times, clearer communication, and the dedicated role of the Case Manager (Table 5).

### 3.5. Challenges and Limitations

Despite its success, the Fast-Track Program faced logistical challenges, especially in maintaining diagnostic slot flexibility during peak periods. Resource constraints, including limited access to FDG-PET and EBUS slots, occasionally delayed the diagnostic process for a subset of patients. Moreover, the program’s dependence on digital communication platforms created minor accessibility barriers for elderly patients.

This structured and patient-centered approach significantly improved diagnostic timelines, adherence to protocols, and patient satisfaction, demonstrating its potential for effective lung cancer diagnosis management, as shown in Table 5.

## 4. Discussion

The Fast-Track Program significantly improved the efficiency of lung cancer diagnostics in the geographically complex Valtellina region. The program led to a 40.5% reduction in the mean time to definitive diagnosis (from 42.9 days to 25 days with *p* < 0.01), while adherence to diagnostic pathways improved from 71% to 92% (*p* < 0.05) and patient satisfaction increased from 64% to 89% (*p* < 0.05). These findings highlight the feasibility of a structured, multidisciplinary approach in optimizing diagnostic workflows, ensuring timely access to care, and reducing unnecessary delays. Importantly, the observed acceleration in diagnostic timelines directly impacted “diagnostic throughput,” as reflected in Table 3. The structured allocation of diagnostic slots ensured that more patients could complete required tests in a shorter timeframe, thereby increasing the overall efficiency of the diagnostic workflow. Although the diagnostic methods remained unchanged, the reduction in scheduling delays allowed for a higher volume of patients to progress through the diagnostic pipeline within a given period. This improvement highlights the role of an optimized workflow management in enhancing throughput, even when technological and procedural factors remain constant. However, the observed increase in confirmed lung cancer cases did not reach a statistical significance (HR = 0.70 and *p* = 0.14), suggesting that while the program effectively streamlined workflows, further refinements in triage and patient selection criteria may be needed to enhance early detection.

The introduction of a Case Manager played a pivotal role in reducing fragmentation, ensuring efficient scheduling and patient coordination across different levels of care. By proactively guiding patients through the diagnostic process, the Case Manager minimized logistical barriers and facilitated adherence to predefined diagnostic pathways. Another critical innovation was the pre-allocation of diagnostic slots for imaging (CT and FDG-PET) and invasive procedures (bronchoscopy and biopsy), which minimized scheduling delays and directly addressed one of the primary bottlenecks in traditional diagnostic pathways. These slots guaranteed priority access for suspected lung cancer cases, reducing the risk of diagnostic dispersion and improving overall workflow efficiency.

The program also incorporated weekly multidisciplinary team (MDT) meetings, enabling real-time discussion of complex cases among thoracic surgeons, oncologists, radiologists, and pathologists. MDT discussions have been widely recognized as key to optimizing cancer care, improving diagnostic accuracy, and ensuring timely decision-making [2,3,4,6]. In our setting, the integration of telemedicine facilitated specialist collaboration despite geographical constraints, aligning with broader trends in digital healthcare [4].

Comparing our findings with the existing literature, structured diagnostic pathways have demonstrated effectiveness in expediting lung cancer diagnosis and improving adherence to clinical guidelines. Studies by Prades et al. [3] and Holtedahl et al. [2] emphasize the role of structured coordination in reducing diagnostic delays. However, unlike urban Fast-Track models—where logistical constraints are less pronounced—our program was explicitly designed to address challenges in a decentralized, resource-limited setting. This distinction is crucial for rural healthcare systems, where healthcare fragmentation and access delays are frequent barriers to timely diagnosis.

A notable strength of the Fast-Track Program is its integration of both structural (pre-scheduled diagnostic slots) and organizational (Case Manager and MDT meetings) solutions to streamline lung cancer diagnostics. While other models primarily focus on rapid referrals, our approach incorporated real-time access to diagnostic resources, ensuring that high-risk patients received expedited workups without unnecessary delays. This pre-scheduling mechanism was particularly valuable in eliminating delays in high-demand imaging procedures such as FDG-PET and EBUS, which are often critical for accurate lung cancer staging and management.

Despite its successes, the program faced several operational challenges. Resource allocation during peak demand periods emerged as a critical issue, particularly for imaging and invasive procedures. While predefined diagnostic slots mitigated most scheduling conflicts, occasional delays persisted. Expanding procedural capacity and optimizing scheduling flexibility could further enhance program efficiency. Additionally, although telemedicine effectively facilitated MDT discussions, some elderly patients encountered difficulties using digital platforms. Enhancing technical support and patient education on digital communication tools may improve engagement and adherence.

An important consideration is the triage efficiency of the Fast-Track model. While it optimized workflow efficiency, it did not significantly increase the proportion of confirmed lung cancer cases. This suggests that while the program effectively streamlined existing pathways, additional refinements—such as integrating AI-based risk stratification—could enhance case selection and early detection rates. Recent studies have highlighted the role of AI in automating lung cancer subtype classification and risk assessment [13], indicating potential future applications for AI-driven decision support in refining triage processes.

The study’s short four-month observation period limits its ability to assess long-term clinical outcomes, including survival benefits. While reducing diagnostic delays is a critical first step, future studies with extended follow-up are needed to evaluate whether faster diagnosis translates into improved survival and treatment effectiveness. Additionally, the single-center setting and relatively small sample size limit the generalizability of findings. Future multi-center studies with randomized methodologies would provide stronger evidence of the program’s broader applicability.

Beyond lung cancer, the Fast-Track model could be extended to other oncological and chronic diseases requiring structured diagnostic workflows. Conditions such as breast and colorectal cancer, as well as chronic diseases like COPD and heart failure, share similar diagnostic complexities and could benefit from predefined diagnostic pathways and enhanced care coordination [3,6]. Furthermore, structured Fast-Track programs have been successfully implemented in other healthcare settings, demonstrating potential benefits for improving early diagnosis and multidisciplinary care coordination in diverse medical conditions [2,4].

Several broader considerations also warrant attention. Lead-time bias remains a concern in Fast-Track programs, as earlier diagnosis does not inherently improve survival. While this study confirmed a significant acceleration in histological confirmation, its true impact on long-term prognosis remains unclear. Extended follow-up periods are necessary to assess whether diagnostic acceleration translates into survival benefits [12,13,14,15,16]. Additionally, structured Fast-Track models carry a risk of over-referral, increasing patient evaluations without a corresponding rise in confirmed malignancies. While over-referral rates remained low in this study, further refinement of the triage criteria could enhance efficiency and reduce unnecessary investigations [6].

Finally, the psychological impact of Fast-Track diagnostics must be considered. While reduced waiting times generally improve patient satisfaction, the accelerated process may increase anxiety in some individuals, particularly those undergoing multiple diagnostic tests before reaching a definitive diagnosis [17,18]. Patients diagnosed with benign conditions may experience unnecessary emotional distress due to intensive workups, reinforcing the need for enhanced psychological support and patient education throughout the diagnostic process. Integrating patient-reported outcome measures (PROMs) into program evaluations could provide valuable insights into the emotional and quality-of-life impact of accelerated diagnostic pathways.

In conclusion, the Fast-Track Program represents a scalable and adaptable model for improving lung cancer diagnostics in complex healthcare settings. Its success underscores the importance of structured coordination, multidisciplinary collaboration, and dedicated resource allocation in optimizing diagnostic efficiency. Future refinements in triage strategies, digital health integration, and AI-assisted diagnostics could further enhance its effectiveness, while extended follow-up studies will be essential to assess its long-term impact on patient outcomes and healthcare resource utilization.

## 5. Conclusions

The Fast-Track Program successfully optimized lung cancer diagnostics, addressing key logistical challenges in the Valtellina region and demonstrating statistically significant improvements in diagnostic timelines and care coordination. Key innovations, including the rotational Case Manager, predefined diagnostic slots, and structured MDT meetings, enabled timely care while minimizing redundancies and delays.

Despite its success, the study had some limitations, including a small cohort, localized scope, and lack of a randomized control group. The short observation period (four months) also restricted the ability to assess long-term clinical outcomes and survival benefits. Further research is needed to validate the model’s broader applicability and impact on patient care and resource utilization.

The Fast-Track Program represents a scalable model for improving oncological and chronic disease pathways, emphasizing patient-centered solutions, technological integration, and multidisciplinary collaboration. By addressing the needs of rural and resource-limited settings, it enhances diagnostic efficiency and establishes a foundation for more equitable healthcare delivery.

In summary, the Fast-Track Program exemplifies how structured, multidisciplinary interventions can overcome systemic healthcare barriers and improve diagnostic efficiency. Future multi-center studies with extended follow-up periods will be essential to fully assess the long-term clinical and economic impact of the Fast-Track Program. Additionally, evaluating its cost-effectiveness and adaptability to different healthcare settings could further support its integration into routine clinical practice, ensuring optimized diagnostic pathways for lung cancer and other complex diseases.

## Figures and Tables

**Table 1:** Baseline characteristics of the study population.

Characteristics *	Control Group (n = 59)							Fast-Track Group (n = 53)								*p* ^§^
Age (years, mean ± SD)	70.0 ± 11.5							68.5 ± 10.6								0.54
Gender (M/F)	39/20							28/25								0.22
Histological type	NSCLC ADK 23		NSCLC SCC 9	SCLC 5	Mai 9	Ben 2	NA 11	NSCLC ADK 19		NSCLC SCC 8	SCLC 3		Mai 9	Ben 4	NA 10	0.99
Stage (TNM 8° edition)	I 5	II 4	III 11	IV 25	NA 13			I 10	II 2	III 9	IV 17		NA 15			0.67
ECOG PS	0 4	1 31	2 9	3 2	4 1	NA 12		0 7		1 30	2 11	3 0	4 0		NA 5	0.73
Outpatient (O) vs. Inpatient (I)	O = 38 I = 27							O = 36 I = 17								0.64
Brain metastases (No/Yes)	59/0							51/2								0.32
Neurological symptoms (No/Yes)	55/4							52/1								0.71
Respiratory failure (No/Yes)	46/13							47/6								0.43

* Parameters were assessed at the time of the first suspicion of lung cancer. ^§^ Statistically significant with α = 0.05. Abbreviations: NSCLC ADK: non-small cell lung cancer adenocarcinoma; NSCLC SCC: non-small cell lung cancer squamous cell carcinoma; SCLC: small cell lung cancer, Mal: other malignant histologies, Ben: benign histologies; ECOG PS: Eastern Cooperative Oncology Group performance status; NA: Not available/Not assessed.

**Table 2:** Clinical-radiological scenarios and diagnostic pathways: classification of patients into four clinical-radiological scenarios with corresponding standardized diagnostic pathways in the Fast-Track Program.

Scenario	Predefined Diagnostic Pathway	Tests and Procedures Involved
Lymphadenopathy	FNAB/CT-guided biopsy	Cytology/histology
Pleural effusion	Thoracentesis or pleuroscopy with biopsy	Cytology and pleural biopsy
Endoscopically accessible lesions	Bronchoscopy or EBUS	Histological examination
Complex lesions	Advanced imaging (CT, FDG-PET) or surgical biopsy (VATS)	Definitive histology

**Table 3:** Efficiency indicators and endpoints of the Fast-Track Program: indicators used to evaluate the efficiency of the Fast-Track Program compared to the control group, with corresponding *p*-values indicating statistical significance (α = 0.05).

Indicator	Control Group	Fast-Track Program	Target	*p*-Value
Average access time (days)	14	5	≤7 days	0.0001
Total program time (days)	42	25	≤30 days	0.0001
Diagnostic throughput (%)	49	95	≥90%	0.0001

**Table 4:** Efficiency indicators and endpoints used to measure the performance of the Fast-Track Program, including their definitions and the predetermined levels of statistical and clinical significance.

Parameter	Definition	Project Objective
Access time	Time elapsed between the first suspected radiological diagnosis of lung cancer and the activation of the pathway by the Case Manager.	-
Throughput time	Time elapsed between the activation of the pathway by the Case Manager and the cytopathological/histopathological diagnosis of lung cancer, or the decision not to proceed with tissue sampling (e.g., in unfit patients).	-
Total program time (primary endpoint)	Sum of Access Time + Throughput Time, or the time between the suspected diagnosis of lung cancer and the cytopathological/histopathological diagnosis, or the decision not to proceed with tissue sampling.	- ≤20 days for at least 60% of new diagnoses and re-diagnosed recurrences. - ≤40 days for 95% of incident cases.
Time to treatment	Time elapsed between the suspected diagnosis of lung cancer and the initiation of specific therapy.	-
Diagnosis incidence rate (secondary endpoint)	Ratio/hazard ratio (HR) of the incidence of pathological diagnoses of new lung cancer cases and re-diagnosed recurrences during the Fast-Track Program period compared to the observed incidence during the control period.	HR ≥ 1.10 with *p*-value < 0.05 will be considered a significant increase in incidence, indicating a consequent rise in activity volume.

**Table 5:** Diagnostic times, outcomes, and satisfaction in the Fast-Track Program vs. control group: comparison of diagnostic times, adherence to diagnostic pathways, and patient satisfaction between the Fast-Track Program and the control group, with differences expressed as percentages and corresponding *p*-values.

Indicator	Control Group	Fast-Track Program	Difference (%)	*p*-Value
Average time to diagnosis (days)	42	25	−41%	<0.01
Diagnosis within 20 days	15	55	+267%	<0.01
Diagnosis within 40 days	49	95	+94%	<0.01
Complete diagnosis (%)	49	95	+46%	<0.01
Adherence to diagnostic pathways (%)	71	92	+21%	<0.05
Patient satisfaction (“very good” or “excellent”, %)	57	90	+37%	<0.05

**Table 6:** Program time and incidence rate of diagnosis in the Fast-Track vs. control group: Comparison of program time and diagnostic incidence rate between the Fast-Track Program and the control group, with statistical significance and clinical significance assessment.

Parameter	Control Group (n = 59)	Fast-Track Program (n = 53)	*p*-Value	Clinical Significance
Program Time (mean, days)	42.9 (95% CI [35.6–50.3])	25.0 (95% CI [20.8–29.3])	0.0001 ^§^	Not clinically significant
Diagnosis within 20 Days (%)	32%	53%	-	-
Diagnosis within 40 Days (%)	49%	81%	-	-
Diagnostic Incidence Rate (patients/day)	0.56	0.39	0.14	Not clinically significant
Hazard Ratio (HR)	0.70	-	-	-

Key Points: The program time in the Fast-Track group was significantly shorter than in the control group (*p* = 0.0001). Although the observed improvement did not meet predefined clinical significance criteria, it showed a favorable trend. No significant increase in the incidence rate of new diagnoses was observed in the Fast-Track group compared to the control group. Notes: ^§^ Statistically significant with α = 0.05. Abbreviations: HR = Hazard Ratio (rate ratio between the diagnostic incidence rates in the two groups).

## Data Availability

The data supporting the findings of this study are not publicly available due to Italian privacy law (GDPR). However, anonymized data can be made available upon reasonable request from the corresponding author.
